# On the early and developed stages of surface condensation: competition mechanism between interfacial and condensate bulk thermal resistances

**DOI:** 10.1038/srep35003

**Published:** 2016-10-10

**Authors:** Jie Sun, Hua Sheng Wang

**Affiliations:** 1 Institute of Engineering Thermophysics, Chinese Academy of Sciences, Beijing 100190, China; 2 School of Engineering and Materials Science, Queen Mary University of London, London E1 4NS, UK

## Abstract

We use molecular dynamics simulation to investigate the early and developed stages of surface condensation. We find that the liquid-vapor and solid-liquid interfacial thermal resistances depend on the properties of solid and fluid, which are time-independent, while the condensate bulk thermal resistance depends on the condensate thickness, which is time-dependent. There exists intrinsic competition between the interfacial and condensate bulk thermal resistances in timeline and the resultant total thermal resistance determines the condensation intensity for a given vapor-solid temperature difference. We reveal the competition mechanism that the interfacial thermal resistance dominates at the onset of condensation and holds afterwards while the condensate bulk thermal resistance gradually takes over with condensate thickness growing. The weaker the solid-liquid bonding, the later the takeover occurs. This competition mechanism suggests that only when the condensate bulk thermal resistance is reduced after it takes over the domination can the condensation be effectively intensified. We propose a unified theoretical model for the thermal resistance analysis by making dropwise condensation equivalent to filmwise condensation. We further find that near a critical point (contact angle being ca. 153°) the bulk thermal resistance has the least opportunity to take over the domination while away from it the probability increases.

When a vapor is in contact with a solid surface at some temperature below the saturation temperature of the vapor, the vapor condenses to liquid on the surface, releasing to the surface the energy difference between the vapor and liquid states. The condensation mode is conventionally categorized as either dropwise or filmwise depending on the surface wettability. For a long time being, the general understanding of surface condensation has been acquired that dropwise condensation (DWC) and filmwise condensation (FWC) are two contrary modes and the former holds an order of magnitude more efficient in heat transfer than the latter^1^,^2^. The explanation has been given two-fold as: (1) the condensate bulk thermal resistance has been regarded as the crucial factor that determines the overall heat transfer performance and (2) the drop-shedding due to gravity makes DWC superior to FWC in reduction of condensate bulk thermal resistance. This understanding has been verified through plenty of experiments at conventional spatial and temporal scales. However, with the modern computer simulation methods^3^,^4^,^5^,^6^,^7^, cutting-edge experimental resorts^8^,^9^,^10^,^11^,^12^,^13^ and fast-developing nanomachining technologies^14^,^15^,^16^, the microscopic, even molecular-level, phenomena, characteristics and dynamics become observable, manipulatable and customizable. It has been demonstrated that the surface wettability, essentially determined by the solid-fluid interaction, dominates various interfacial phenomena, e.g. velocity slip^6^,^7^, temperature jump^6^,^7^, liquid layering^11^,^12^,^13^, contact angle^3^,^9^,^17^ and droplet jumping^8^,^15^,^18^. Therefore, the fundamental understanding of surface condensation needs to be elucidated by molecular-level insights.

Our recent work^3^, focused on the onset of surface condensation by molecular dynamics simulation and classical nucleation theory, has revealed the intrinsic connection between DWC and FWC. The solid-fluid interaction has been found as the origin to determine the surface condensation mode from the onset. A whole picture of condensation mode on surface with a wide range of wettability has been given, where DWC is taken as the transition mode between no-condensation and FWC. In this work, we continue to explore the insight of surface condensation after the onset, i.e. the early and developed stages. We transform DWC equivalent to FWC in terms of condensate bulk thermal resistance, enabling unified analysis of the complex phenomena. We reveal that the competition between the interfacial and condensate bulk thermal resistances determines the characteristics of time evolutions of different condensation modes and further the condensation intensities. The formation and transition mechanisms revealed in our previous work^3^ presents the spatial characteristics of unification of surface condensation while the competition mechanism revealed in this work presents its temporal characteristics. These characteristics clarify a fundamental insight of the phenomenon of surface condensation.

## Results

**Figure 1:**
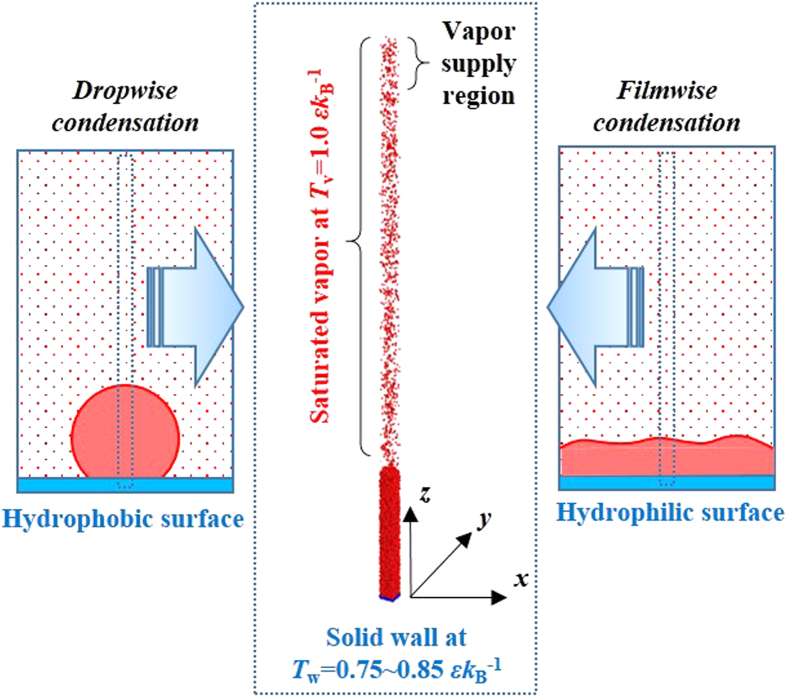
Schematic of simulation system for surface condensation. The simulation size measures 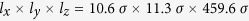. The saturated vapor (red) is at 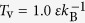. The solid wall (blue) is at the bottom and is at 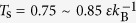. The periodic boundary condition is applied at the sides and the diffuse reflection boundary is applied at the top end. The vaper supply region (thickness 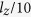) is at the top. The surface wettability of solid wall changes from hydrophobicity to hydrophilicity by varying *β* from 0.1 to 0.7.

**Figure 2:**
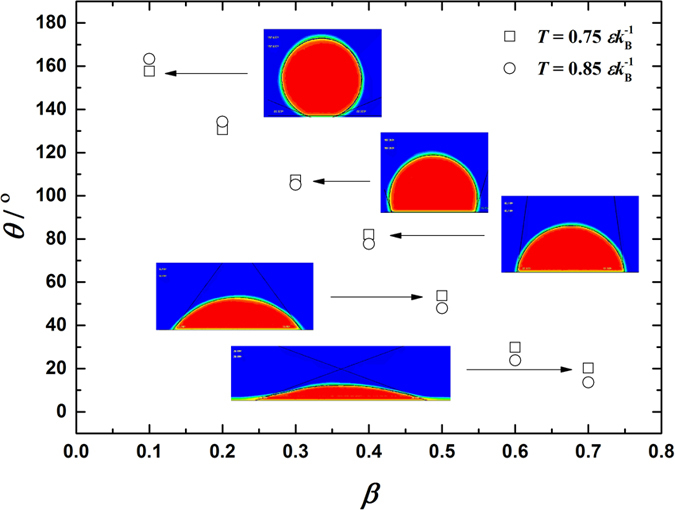
Correlations of contact angle (*θ*) with fluid-solid bonding parameter (*β*). The simulation size measures 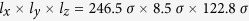. The simulations are carried out in thermodynamic equilibrium state at 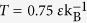 and 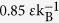 with 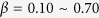. The insets are for 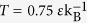.

We use molecular dynamics (MD) simulation to characterize the early and developed stages of surface condensation. A long cuboid simulation box is chosen to take the general feature that affect both DWC and FWC at these stages and to eliminate the vapor-liquid-solid contact-line of DWC (see Fig. 1). The fluid-fluid interaction is governed by the Lennard-Jones (L-J) potential function. The fluid-solid interaction is also described by the L-J potential function, where the parameter *β* measures the relative strength of fluid-solid bonding. A small value of *β* means low surface free energy and hydrophobicity while a large value of *β* means higher surface free energy and hydrophilicity^6^,^7^,^19^,^20^,^21^,^22^,^23^. The surface wettability is commonly interpreted by contact angle *θ*, a readily measureable quantity^24^,^25^,^26^. To establish the correlation between *β* and *θ*, we primarily conduct MD simulations at thermal equilibrium states of 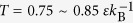 (see Fig. 2), *k*_B_ being the Boltzmann constant. The results show that *θ* decreases monotonically from 160° to 20° corresponding to *θ* from 0.1 to 0.7.

**Figure 3:**
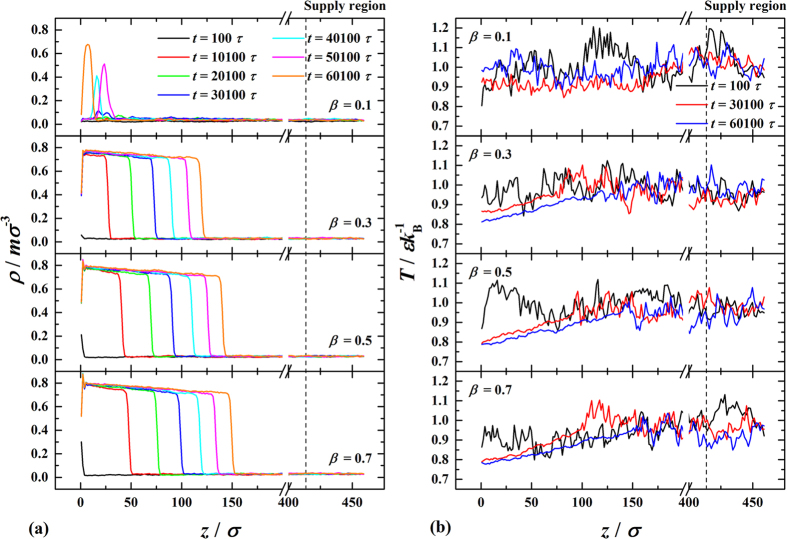
Variations of the profiles of (**a**) density and (**b**) temperature in *z*-direction at different times (*t*) in the condensation period (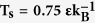).

We perform MD simulations for various values of fluid-solid bonding strength under non-isothermal conditions that the solid temperature varies in the range of 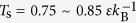 and the saturated vapor temperature is fixed at 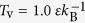. The transient density profiles (see Fig. 3a) show that on the surface with *β* = 0.1, where the fluid-solid interaction is very weak, the condensate bulk can hardly form. On the contrary, the condensate bulk is seen to form readily on the surface with *β* = 0.7, where the fluid-solid interaction is very strong, and to keep growing thicker with time, indicating a high condensation intensity. Generally, the condensate bulk grows faster for higher *β* but the growth decreases with time. The transient temperature profiles (see Fig. 3b) show that with the condensate bulk forming on surface, a local temperature gradient establishes spontaneously within the condensate bulk connecting the solid-liquid and liquid-vapor interfaces. The range of the locally linear temperature profile widens with increasing thickness of the condensate bulk. During the widening, the local temperature at the solid-liquid interface gradually decreases approaching the surface temperature *T*_s_ while the local temperature at the liquid-vapor interface basically holds close to the vapor bulk temperature *T*_v_. Note that the locally linear temperature profile fails to establish for *β* = 0.1 but behaves more regularly for higher *β*. Generally, the temperature gradient increases with increasing *β* but decreases with time. Both the density and temperature profiles suggest that the condensation intensity becomes higher with increasing *β* but lower with time.

**Figure 4:**
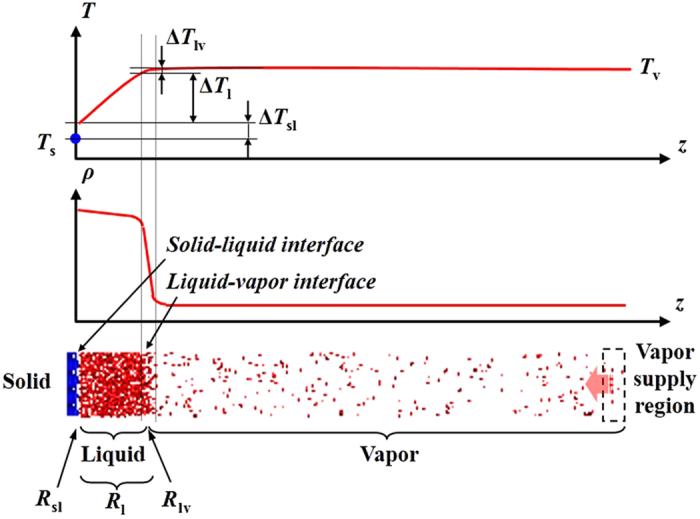
Schematic presentation of interfacial and condensate bulk thermal resistances.

As shown in Fig. 4, the condensation heat flux (*q*_c_) originates from the vapor bulk distant from the condensing surface. With the vapor molecules approaching and condensing into the condensate bulk, the energy difference between the vapor and liquid states is released at the liquid-vapor interface. *q*_c_ then flows through the condensate bulk and across the solid-liquid interface via pure heat conduction (see linear temperature profile in Fig. 3b). Each step comes with a thermal resistance against *q*_c_. Therefore, the overall thermal resistance (*R*_total_) consists of the liquid-vapor interfacial thermal resistance (*R*_lv_), the condensate bulk thermal resistance (*R*_l_) and the solid-liquid interfacial thermal resistance (*R*_sl_). Correspondingly, there are temperature drops from vapor to solid Δ*T*, namely, Δ*T*_lv_, Δ*T*_1_ and Δ*T*_sl_. Essentially, the characteristics of density and temperature profiles are closely related to the thermal resistances. The uniformity in both density and temperature profiles indicates the region of vapor bulk. The sudden drop in density profile corresponds to *R*_lv_, the local linearity in density and temperature profiles corresponds to *R*_1_ and the local temperature jump corresponds to *R*_sl_. For a given vapor-to-solid temperature difference (Δ*T*), *q*_c_ is determined as:


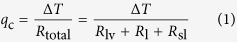


Since *q*_c_ holds through the thermal resistances, then we have


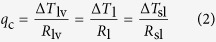


**Figure 5:**
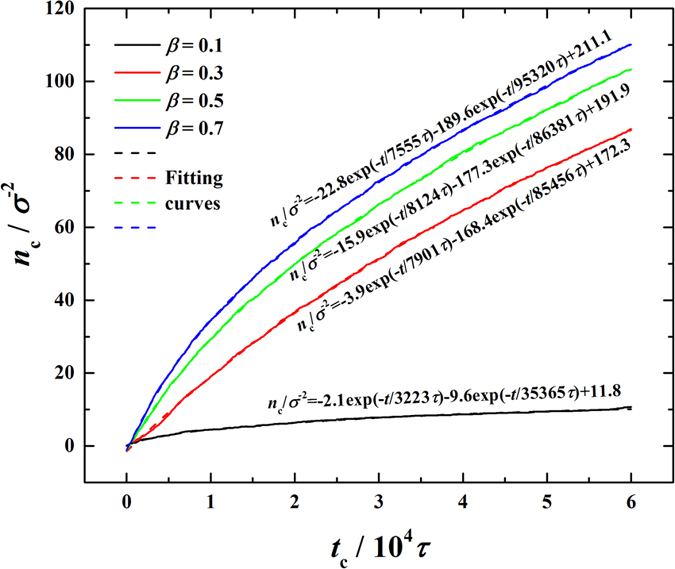
Time evolutions of number of condensed molecules (*n*_c_) in the condensation period (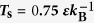).

**Figure 6:**
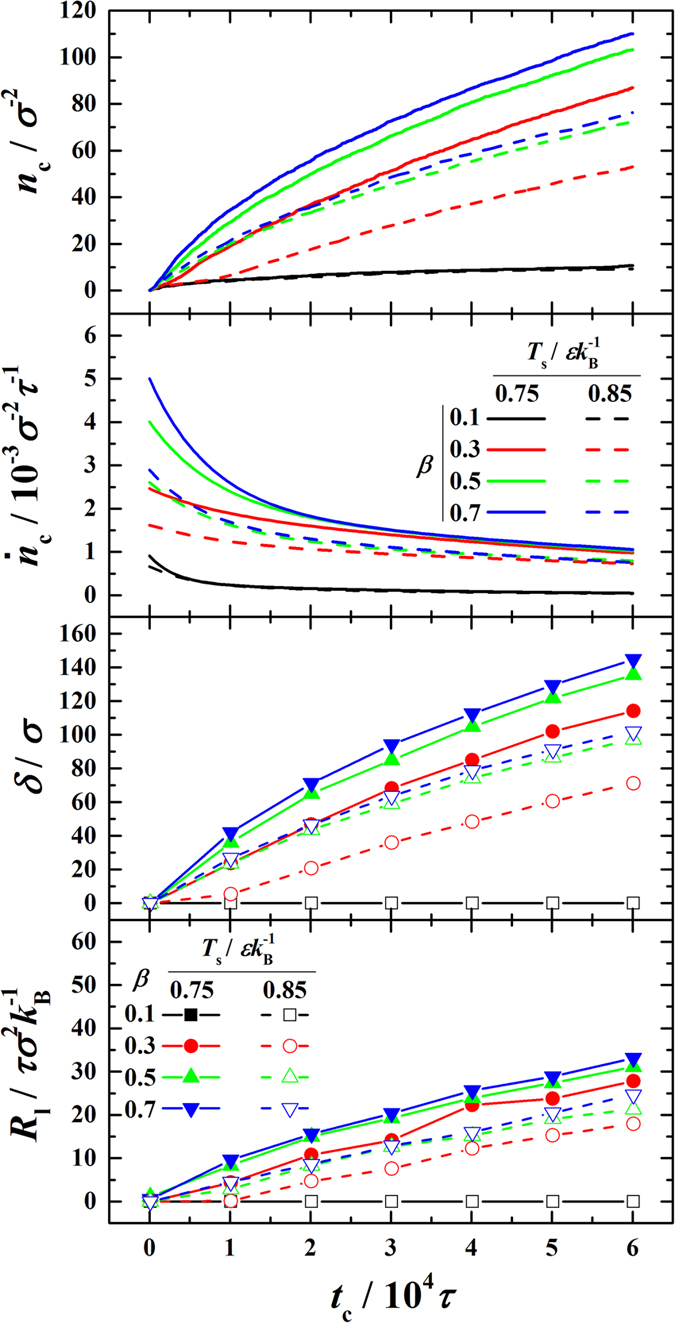
Time evolutions of number of condensed molecules (*n*_c_), condensation flux (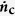), thickness of condensate (*δ*) and condensate bulk thermal resistance (*R*_l_) in the condensation period.

In this work, Δ*T* is calculated as:


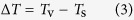


and *q*_c_ is calculated as:


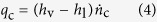


where *h*_v_ and *h*_1_ are the specific enthalpies in vapor and condensate bulks, respectively (see details in ref. 27); 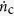 is the condensed molecule flux, i.e. the time derivative of *n*_c_ (the number of condensed molecules per unit area). In the simulations, we carefully monitor the time evolution of *n*_c_ and find that *n*_c_ perfectly follows a second-order exponential increasing function, as:


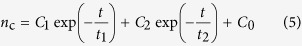


where *C*_0_, *C*_1_, *C*_2_ and *t*_1_, *t*_2_ are all coefficients. For demonstration, the time evolutions of *n*_c_ for the case of 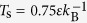 and corresponding fitting curves are given in Fig. 5, from which we can clearly see that *n*_c_ grows decreasingly with time, fitting the second-order exponential increasing function, and increases with increasing *β*. 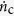 is obtained by differentiation, as:


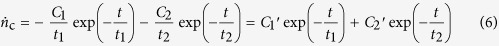


where 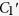 (=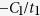) and 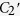 (=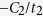) are coefficients. Apparently, 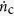 follows a second-order exponential decreasing function. The time evolutions of *n*_c_ and 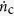 are shown in the first and second panels of Fig. 6, from which we find that, for a same *β*, the condensation intensity increases with increasing Δ*T* as expected.

The thickness of condensate bulk (*δ*) grows with condensation ongoing. For quantification, the liquid-side boundary of liquid-vapor interface is taken as the surface of the condensate bulk, which is the same as those in refs 28,29. We have tried both the density-based method^30^ and SRK equation-based method^28^ to determine the surface position and find that the results agree well with each other. The time evolutions of *δ* are shown in the third panel of Fig. 6.

**Figure 7:**
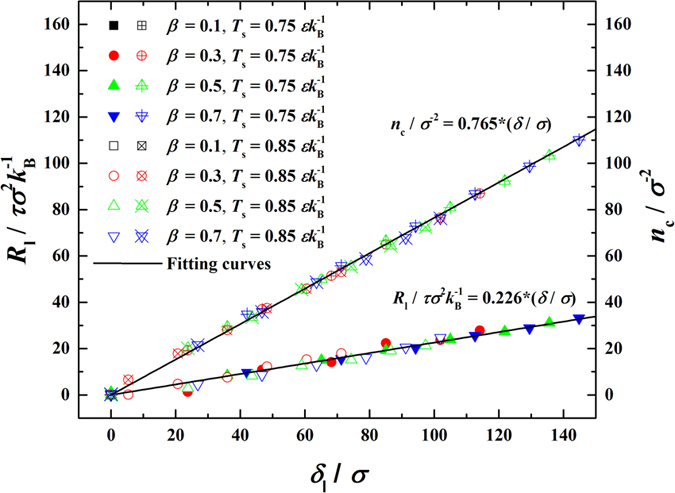
Correlations of condensate bulk thermal resistance (*R*_l_) and number of condensed molecules (*n*_c_) with thickness of condensate bulk (*δ*) in the condensation period.

**Figure 8:**
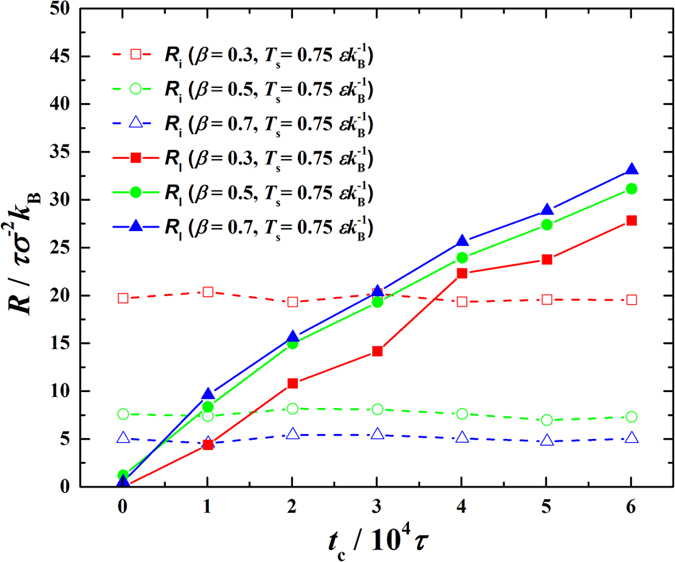
Time evolutions of interfacial thermal resistance (*R*_i_) and condensate bulk thermal resistance (*R*_l_) in the condensation period.

Since *q*_c_ can be calculated by Eq. (4) and Δ*T*_lv_, Δ*T*_l_ and Δ*T*_sl_ can be determined directly from the temperature profiles (see Fig. 3b), consequently, *R*_lv_, *R*_l_ and *R*_sl_ are readily obtained based on Eq. (2). With condensation ongoing, *R*_l_ apparently increases with increasing *δ*, as shown in the lowermost panel of Fig. 6. By correlating *R*_1_ and *n*_c_ to *δ*, we confirm 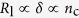 through that all data of *R*_1_ and *n*_c_ fall well in linear functions of *δ* (see Fig. 7). It is implied that *R*_1_ and *δ* are also of the second-order exponential increasing functions of time as is *n*_c_. On the other hand, *R*_lv_ is supposed to be solely dependent on the thermodynamic state of fluid and 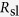 is known to be solely dependent on *β*. Therefore, for a given Δ*T*, the interfacial thermal resistances (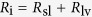) essentially remain constant. In the simulations, *R*_1v_ is estimated to be 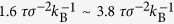 for 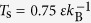 and 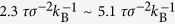 for 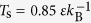 while *R*_sl_ is estimated to be 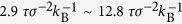 for 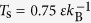 and 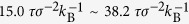 for 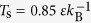. Clearly, *R*_lv_ is much smaller than 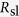. For demonstration, the time evolutions of *R*_i_ and *R*_l_ for 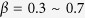 and 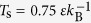 are shown in Fig. 8. We can clearly see the competition between *R*_i_ (the time-independent component) and *R*_l_ (the time-dependent component) over time. At the initial period of time, *R*_i_ undoubtedly dominates in *R*_total_ and holds while with condensation ongoing *R*_l_ gradually increases, surpasses *R*_i_ and finally takes over the domination in *R*_total_. Note that the condensation intensity is so low for *β* = 0.1 that *R*_i_ is not applicable and *R*_1_ keeps zero therefore these are not shown. By comparing Figs 6 and 8, we find that the moments *R*_l_ surpassing *R*_i_ are basically the inflection points in the time evolutions of 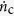. Apparently, the initially fast decreasing part of 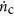 is dominated by *R*_i_ while the afterwards slow decreasing part is dominated by *R*_l_. Since *R*_i_ is due to inherent properties of solid and fluid, which cannot be changed during condensation, while *R*_l_ is linearly correlated to *δ*, which is artificially removable during condensation, it is concluded that the condensation could be effectively intensified by removing *R*_l_ out of *R*_total_ only when *R*_l_ has defeated *R*_i_. This provide clear guidance on how to enhance condensation heat transfer using highly-customized nano-machining and nano-coating surfaces^31^,^32^,^33^.

Based on the above results that *R*_l_ grows as a second-order exponential increasing function of time while *R*_i_ is independent of time, we can finally achieve the following correlation from Eq. (1), as:


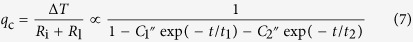


where 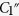 and 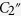 are coefficients. This indicates that *q*_c_ is supposed to be an inversed second-order exponential decreasing function of time.

**Figure 9:**
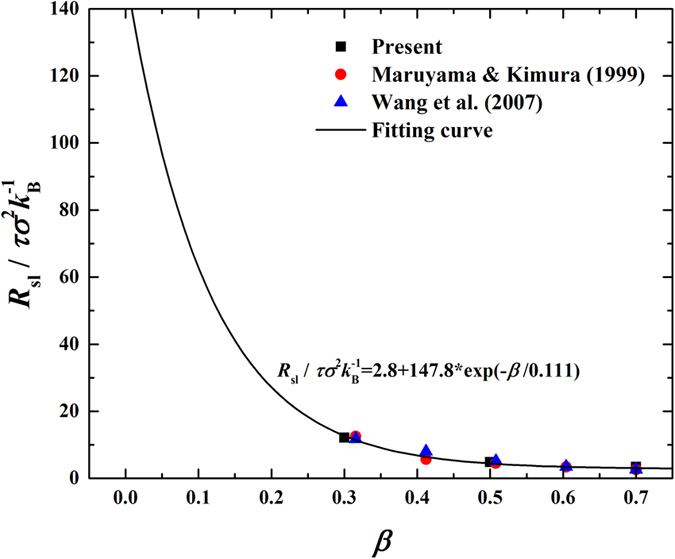
Correlation of solid-liquid interfacial thermal resistances (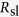) with fluid-solid bonding parameter (β).

In addition, it is noteworthy that *R*_sl_ has been known to be correlated to *β* as a monotonically and nonlinearly decreasing function^21^,^34^,^35^. Our estimation of *R*_sl_ agrees well with the published data and we further successfully fit all the data-points into an exponential decreasing function (see Fig. 9).

## Discussion

**Figure 10:**
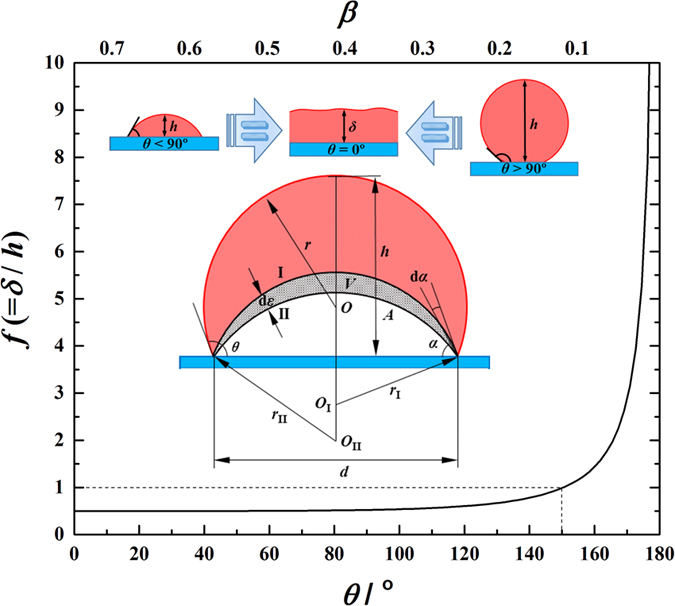
Equivalent ratio (*f* = *δ*/*h*) against contact angle (*θ*).

The simulation system in this work can be used to directly analogize FWC with one-dimensional characteristics but not DWC, with inherent three-dimensional characteristics. To reasonably carry out a theoretical analysis of thermal resistance between FWC and DWC, we propose a unified model, where the condensate bulk thermal resistance of DWC is made equivalent to that of FWC (see upper inset in Fig. 10). For the conductive heat transfer through the condensate bulk with a temperature difference Δ*T*_1_, the general equation has the form 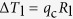. For FWC, 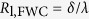 therefore 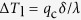 (*λ* being the thermal conductivity of condensate bulk and taken to be constant for simplification). For DWC, we normally have 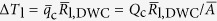, where 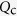, 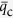, 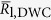 and 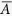 are the condensation heat transfer rate, equivalent condensation heat flux, equivalent condensate bulk thermal resistance and equivalent heat transfer area, respectively. Based on the ideally spherical crown for a single droplet on solid surface (see lower inset in Fig. 10), the surfaces I and II are the isothermal surfaces and the distance (d*ε*) between the two surfaces is the local thickness of the condensate bulk thermal resistance. The average value of d*ε* is obtained as:


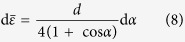


where *d* is the diameter of the contact circle. Therefore, 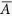 is calculated by the integral mean value of *A*, as:


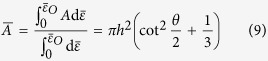


where *h* is the height of the spherical crown. Note that 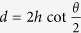. On the other hand, for DWC, Δ*T*_1_ can be directly obtained by integrating the differential form of Fourier’s equation of heat conduction, as


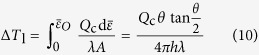


We now have the expression for 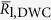 as


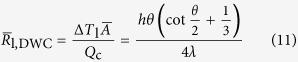


If we let 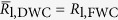, i.e. 
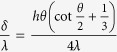
, and we define an equivalent factor *f* (=*δ*/*h*), then we have


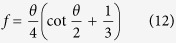


That means DWC with the height of *h* can be equivalently treated as FWC with the thickness of *δ* when Eq. (12) is satisfied. All the details of calculus are given in Supplementary Information. The curve of Eq. (12) is drawn in Fig. 10. We find that the curve starts from *f* = 0.5 at *θ* = 0° and then gradually increases with increasing *θ* (decreasing *β*) until it approaches around *θ* = 160°, after which it suddenly starts to grow increasingly towards infinity at *θ* = 180°. Note that *f* = 1 corresponds to *θ* ≈ 150°. This variation indicates that (1) the condensate bulk thermal resistance of a droplet with *θ* = 0° is equivalent to that of the condensate film with the thickness only half the height of the droplet (*f* = 0.5); (2) the condensate bulk thermal resistance of a droplet with *θ* = 180° is equivalent to that of the condensate film with infinite thickness (*f* → ∝); (3) the condensate bulk thermal resistance of a droplet with *θ* ≈ 150° is equivalent to that of the condensate film with the thickness being equal to the height of the droplet (*f* = 1); (4) the equivalent factor increases nonlinearly with increasing *θ*. Note that *θ* and *β* are not strictly linear to each other.

**Figure 11:**
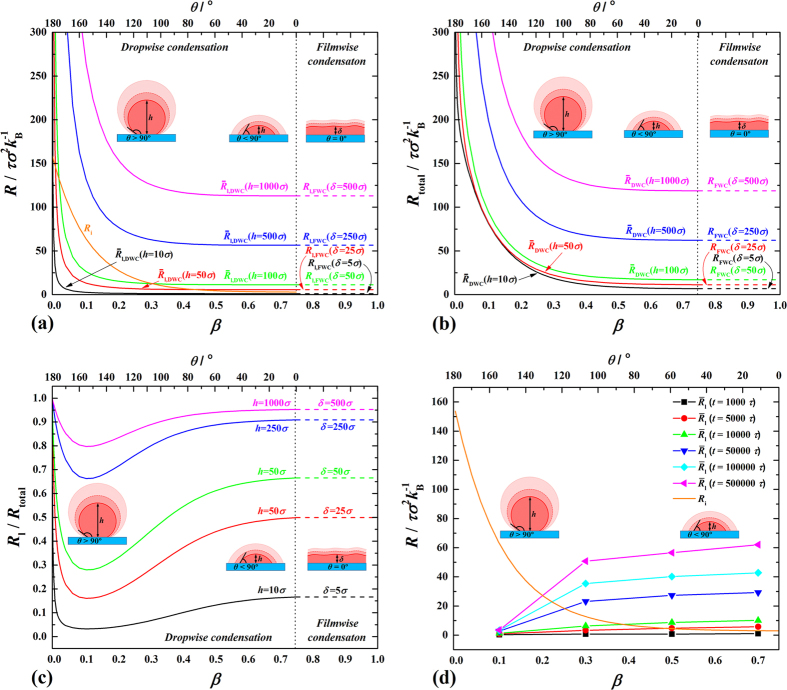
Analytical results of surface condensation. (**a**) Competition between interfacial thermal resistance (*R*_i_) and condensate bulk thermal resistance (*R*_l_) against fluid-solid bonding parameter (*β*) and *θ*; (**b**) Total thermal resistance (*R*_total_) against *β* and *θ*; (**c**) Ratio of *R*_l_ to *R*_total_ against *β* and *θ*; (**d**) Transient variations of *R*_l_ and *R*_i_ against *β* and *θ* (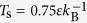).

The curves of 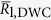, 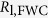 and 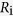 (see Fig. 11a) show that 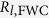 is solely dependent on *δ* while 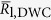 is dependent on both *h* and *β*. 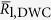 reduces decreasingly with increasing *β* and with an appreciable inflection point, which increases with increasing *h*. When *h* is small, e.g. 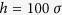, there is a range of 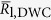 below *R*_i_, e.g. *β* = 0.017∼0.3, meaning the latter overcomes the former. This range shrinks with increasing *h*. When *h* is large enough, e.g. 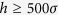, 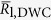 is no longer below *R*_i_ at any value of *β*, meaning the former takes the absolute domination regardless of condensate shape or condensation mode. The curves of *R*_total_ (see Fig. 11b) basically shape the same way as in Fig. 11a because *R*_l_ and *R*_i_ constitute the overwhelming majority of *R*_total_. Note that for FWC, *R*_total_ only includes *R*_sl_, *R*_l_ and *R*_lv_, while for DWC, a curvature-induced thermal resistance (*R*_curv_) exists and is estimated as 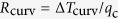, where Δ*T*_curv_ is calculated as^36^:


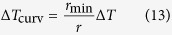



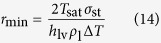


where *T*_sat_ is the saturation temperature, *σ*_st_ is the surface tension at *T*_sat_, 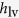 is the latent heat and *ρ*_1_ is the condensate bulk density. In this work, *r*_min_ is estimated to be 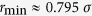, which means that when 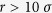 the relative influence of *R*_curv_ is less than 8%. Therefore, the effect of *R*_curv_ can be hardly felt in Fig. 11b. For proportional analysis, the curves of 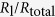 (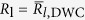 for DWC and 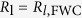 for FWC) are shown in Fig. 11c. We can clearly see that there exists a minimum for all curves corresponding to a common critical value of 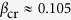 (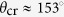). This critical point actually corresponds to *f* = 1 in Fig. 10 with small deviation caused by *R*_curv_. This means that for a given equivalent thickness to FWC, *f* = 1 guarantees the minimal *R*_l_/*R*_total_. The reason is two-sided: (1) When *β* > *β*_cr_ (*θ* < *θ*_cr_), *f* < 1 leads to decreasing *R*_l_ but *R*_total_ decreases due to more decreasing *R*_i_ (see Fig. 10) therefore the resultant *R*_l_/*R*_total_ increases; (2) When 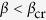 (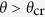), *f* > 1 leads to more increasing *R*_l_ though *R*_toatl_ also increases due to increasing *R*_i_ (see Fig. 10) therefore the resultant *R*_l_/*R*_total_ also increases. This point could shed some light on the design and optimization of superhydrophobic surfaces for DWC, where the so-called self-propelled droplet-jumping (removal of *R*_l_) takes place^15^,^37^,^38^,^39^. Based on the above-acquired second-order exponential correlation of *R*_l_ against time, we show the competition between *R*_l_ and *R*_i_ over time in Fig. 11d. We find that for FWC, *R*_l_ can easily defeats *R*_i_ with time lapse while for DWC, the probability decreases with decreasing *β* (increasing *θ*).

**Figure 12:**
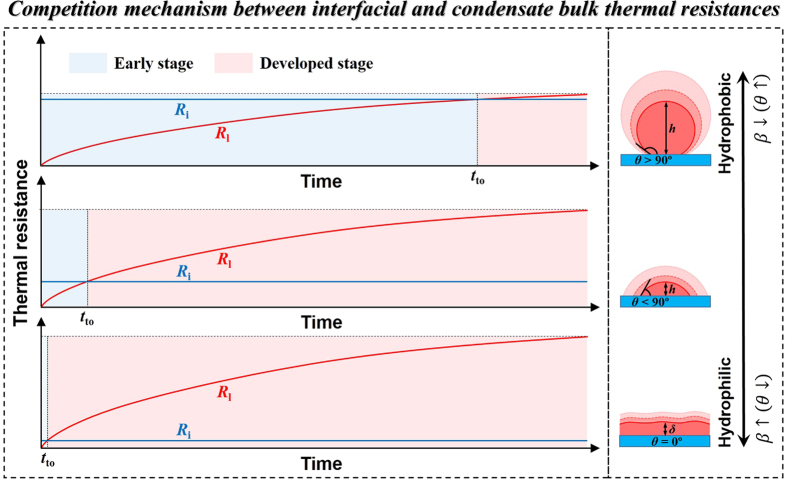
Schematic presentation of the competition mechanism between the interfacial and condensate bulk thermal resistances .

To clearly illustrate our competition mechanism, a schematic presentation is given in Fig. 12. We show the typical evolutions of *R*_l_ and *R*_i_, corresponding to DWC with *θ* > 90°, DWC with *θ* < 90° and FWC (*θ* = 0°), in the timeline. Generally, *R*_l_ grows decreasingly with time in a second-order exponential way while *R*_i_ generally keeps constant. We define the time when *R*_l_ surpasses *R*_i_ as the takeover time (*t*_to_) and use it as the threshold between the early and developed stages. For DWC with *θ* > 90°, the solid-fluid interaction is very weak (small *β*) leading to large *R*_i_. On the other hand, weak solid-fluid interaction hinders the mass and heat transfer hence weakens the condensation intensity leading to slowly increasing *R*_l_. The resultant *t*_to_ is much delayed. For DWC with *θ* < 90°, the solid-fluid interaction is strong (large *β*) leading to small *R*_i_. On the other hand, strong solid-fluid interaction enhances the mass and heat transfer hence strengthens the condensation intensity leading to quickly increasing *R*_l_. The resultant *t*_to_ is advanced. For FWC, the solid-fluid interaction is very strong (larger *β*) leading to tiny *R*_i_. On the other hand, very strong solid-fluid interaction significantly strengthens the condensation intensity leading to more quickly increasing *R*_l_. The resultant *t*_to_ is extremely early. We find that the period of early stage is elongated with decreasing *β*. Note that *β* being extremely small (*θ* → 180°) suggests infinite period of early stage. Since heat transfer with fixed temperature difference can be enhanced by reducing the thermal resistance, we emphasize that with fixed Δ*T* only in the developed stage the condensate is removed can the condensation be effectively enhanced. Otherwise, the heat transfer is still dominated by *R*_i_ and the removal is supposed to be in vain.

We point out that this work is more focused on the early stage of surface condensation when *R*_i_ is yet comparable to *R*_l_. As shown in Fig. 11a, given sufficiently long time, *R*_l_ will defeat all the other thermal resistances and eventually become dominant in *R*_total_. This is the developed stage of surface condensation, where the conventional understanding becomes applicable (see Introduction). We also point out that Fig. 12 provides us a whole picture of the transitional roles of thermal resistances for surface condensation. This work, in line with our recent work on formation and transition mechanisms of condensation mode^3^, further bridges the early stage to developed stage of surface condensation in terms of time evolutions of thermal resistances.

In summary, we use MD simulation to investigate the characteristics of condensate growth in the early and developed stages of surface condensation. We divide the thermal resistances into interfacial and condensate bulk components. The interfacial thermal resistances solely depend on the properties of solid and fluid, which are time-independent. However, the condensate bulk thermal resistance and also the condensate thickness and the number of condensed molecules, which are time-dependent, are all second-order exponential increasing functions of time. The condensation heat flux is supposed to be an inversed second-order exponential decreasing function of time. With condensation ongoing, there exists an intrinsic competition between the interfacial and condensate bulk thermal resistances and the resultant total thermal resistance determines the condensation intensity under a given vapor-solid temperature difference. The competition mechanism works as that the interfacial thermal resistance dominates at the onset of condensation and holds afterwards while the condensate bulk thermal resistance gradually takes over with condensate thickness growing. The takeover time is longer for a smaller *β*. This competition mechanism suggests that only when the condensate bulk thermal resistance is removed after it takes over the domination can the condensation be effectively intensified. Otherwise, the effectiveness could be little. To fairly carry out the thermal resistance analysis, we propose a unified theoretical model by making DWC equivalent to FWC. The theoretical results indicate that near the critical point (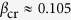, 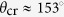), the condensate bulk thermal resistance has the least opportunity to take over the domination while away from it the probability increases. By implementing the correlation of condensate bulk thermal resistance with time, we find that for FWC the condensate bulk thermal resistance can easily defeats the interfacial thermal resistance over time while for DWC the probability decreases with increasing contact angle, i.e. decreasing *β*. We reveal the transitional roles of thermal resistances over time in surface condensations, which provides full understanding of the insight of the surface condensation for different modes over a whole range of conditions and bridges the early stage to developed stage of surface condensation.

## Methods

The fluid-fluid interaction is governed by the Lennard-Jones (L-J) potential function, as:


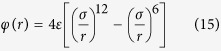


where *r* is the intermolecular separation, *σ* and *ε* are the length and energy scales, respectively^40^. The function is truncated by the cut-off radius 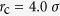, beyond which the interactions are not considered. The fluid-solid interaction is also described by the L-J potential function with length scale 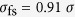 and energy scale 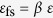, where the parameter *β* measures the relative strength of fluid-solid bonding.

The solid wall, i.e. the condensing surface, is set at the bottom of the simulation box and represented by three layers of solid molecules forming a (111) plane of a face-centered cubic lattice with the lattice constant 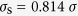. Neighboring solid molecules are connected by Hookean springs with the constant 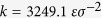 41. Two extra layers of solid molecules are set below the three layers. The lower is stationary as a frame while the upper is governed by the Langevin thermostat, as:


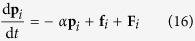


where **p**_*i*_ is the momentum of the *i*th solid molecule; 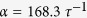 is the damping constant^42^, **f**_*i*_ is the sum of the forces acting on the *i*th solid molecule, **F**_*i*_ is a random force, of which each component is sampled from the Gaussian distribution with zero mean value and variance 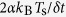 (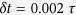 is the time step, where 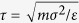 is the time scale, *m* being the mass of a fluid molecule)^41^,^42^. The periodic boundary condition is applied at the sides and the diffuse reflection boundary is applied at the top end.

In each run, a relaxation period of 200 *τ* is used to keep the vapor saturated at 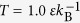, followed by the condensation period of 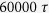 with the solid temperature at a value in the range of 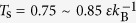 and the saturated vapor temperature at 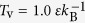, respectively. Extra vapor molecules are supplied through the upmost supply region (thickness *l*_*z*_/10) by the USHER algorithm^43^ immediately when the density within the supply region is lower than its initial saturation value. During the condensation period, the temperature in the supply region is controlled at 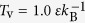 by the Langevin thermostat^20^,^44^. The surface wettability of solid wall changes from hydrophobicity to hydrophilicity by varying *β* from 0.1 to 0.7.

## Additional Information

**How to cite this article**: Sun, J. and Wang, H. S. On the early and developed stages of surface condensation: competition mechanism between interfacial and condensate bulk thermal resistances. *Sci. Rep.* **6**, 35003; doi: 10.1038/srep35003 (2016).

## Supplementary Material

Supplementary Information
