# Supplementary material for: On the early and developed stages of surface condensation: competition mechanism between interfacial and condensate bulk thermal resistances
Source: Sci Rep. 2016 Oct 10;6:35003. doi: 10.1038/srep35003 (PMC5056363; doi:10.1038/srep35003)
Supplement: Supplementary Information [file srep35003-s1.doc]

**On the early and developed stages of surface condensation: competition mechanism between interfacial and condensate bulk thermal resistances**

Jie Sun and Hua Sheng Wang

As shown in the lower insert in FIG. 10, the average distance $\text{d}\bar{\varepsilon}$ between the surfaces I and II can be obtained by dividing the volume of the shaded part *V* by the basal area *A*. The volume of the shaded part equals to the volume difference of the spherical crown $V_{\text{I}}$ subtracted by the spherical crown $V_{\text{II}}$, as:

$$\text{d}\bar{\varepsilon}=\frac{V}{A}=\frac{V_{\text{I}}-V_{\text{II}}}{A}$$

According to geometry, we have

$$\begin{matrix} V_{\text{I}}=\frac{\pi}{3}\left(3r_{\text{I}}-h_{\text{I}}\right)h_{\text{I}}^{2} \\ =\frac{\pi}{3}\left[3r-r_{\text{I}}\left(1-\cos\alpha\right)\right]r_{\text{I}}^{2}\left(1-\cos\alpha\right)^{2} \\ =\frac{\pi r_{\text{I}}^{3}}{3}\left(2+\cos\alpha\right)\left(1-\cos\alpha\right)^{2} \\ =\frac{\pi d^{3}\left(2+\cos\alpha\right)\left(1-\cos\alpha\right)^{2}}{24\sin^{3}\alpha} \end{matrix}$$

$$\begin{matrix} V_{\text{II}}=\frac{\pi}{3}\left(3r_{\text{II}}-h_{\text{II}}\right)h_{\text{II}}^{2} \\ =\frac{\pi}{3}\left\{3r-r_{\text{II}}\left[1-\cos\left(\alpha+\text{d}\alpha\right)\right]\right\}r_{\text{II}}^{2}\left[1-\cos\left(\alpha+\text{d}\alpha\right)\right]^{2} \\ =\frac{\pi r_{\text{II}}^{3}}{3}\left[2+\cos\left(\alpha+\text{d}\alpha\right)\right]\left[1-\cos\left(\alpha+\text{d}\alpha\right)\right]^{2} \\ =\frac{\pi d^{3}\left[2+\cos\left(\alpha+\text{d}\alpha\right)\right]\left[1-\cos\left(\alpha+\text{d}\alpha\right)\right]^{2}}{24\sin^{3}\left(\alpha+\text{d}\alpha\right)} \end{matrix}$$

$$\begin{matrix} A=2\pi r_{\text{I}}^{2}\left(1-\cos\alpha\right) \\ =\frac{\pi d^{2}\left(1-\cos\alpha\right)}{2\sin^{2}\alpha} \\ =\frac{\pi d^{2}}{2\left(1+\cos\alpha\right)} \end{matrix}$$

Note that $r_{\text{I}}=\frac{d}{2\sin\alpha}$ and $r_{\text{II}}=\frac{d}{2\sin\left(\alpha+\text{d}\alpha\right)}$

Considering Eqs. ~, we have

$$\begin{matrix} \text{d}\bar{\varepsilon}=\frac{d\sin^{2}\alpha}{12\left(1-\cos\alpha\right)}\left\{\frac{\left[2+\cos\left(\alpha+\text{d}\alpha\right)\right]\left[1-\cos\left(\alpha+\text{d}\alpha\right)\right]^{2}}{\sin^{3}\left(\alpha+\text{d}\alpha\right)}-\frac{\left(2+\cos\alpha\right)\left(1-\cos\alpha\right)^{2}}{\sin^{3}\alpha}\right\} \\ =\frac{d\sin^{2}\alpha}{12\left(1-\cos\alpha\right)}\left[\frac{\left(2+\cos\alpha\right)\left(1-\cos\alpha\right)^{2}}{\sin^{3}\alpha}\right]^{'}\text{d}\alpha\\ =\frac{d\sin^{2}\alpha}{12\left(1-\cos\alpha\right)}\frac{3\left(\cos\alpha-1\right)^{2}}{\sin^{4}\alpha}\text{d}\alpha\\ =\frac{d\left(1-\cos\alpha\right)}{4\sin^{2}\alpha}\text{d}\alpha\\ =\frac{d}{4\left(1+\cos\alpha\right)}\text{d}\alpha\end{matrix}$$

Therefore, we have the following integrals based on Eqs. and

$$\begin{matrix} \int_{0}^{\bar{\varepsilon}_{O}} A\text{d}\bar{\varepsilon}=\int_{0}^{\theta} \frac{\pi d^{2}}{2\left(1+\cos\alpha\right)}\frac{d}{4\left(1+\cos\alpha\right)}\text{d}\alpha\\ =\frac{\pi d^{3}}{8}\int_{0}^{\theta} \frac{\text{d}\alpha}{\left(1+\cos\alpha\right)^{2}} \\ =\frac{\pi d^{3}}{16}\left.\left(\tan\frac{\alpha}{2}+\frac{1}{3}\tan^{3}\frac{\alpha}{2}\right)\right|_{0}^{\theta} \\ =\frac{\pi d^{3}}{16}\left(\tan\frac{\theta}{2}+\frac{1}{3}\tan^{3}\frac{\theta}{2}\right) \\ =\frac{\pi h^{3}}{2}\left(\cot^{2}\frac{\theta}{2}+\frac{1}{3}\right) \end{matrix}$$

$$\begin{matrix} \int_{0}^{\bar{\varepsilon}_{O}} \text{d}\bar{\varepsilon}=\int_{0}^{\theta} \frac{d}{4\left(1+\cos\alpha\right)}\text{d}\alpha\\ =\frac{d}{4}\int_{0}^{\theta} \frac{1}{\left(1+\cos\alpha\right)}\text{d}\alpha\\ =\frac{d}{4}\left.\tan\frac{\alpha}{2}\right|_{0}^{\theta} \\ =\frac{d}{4}\tan\frac{\theta}{2} \\ =\frac{h}{2} \end{matrix}$$

$$\begin{matrix} \Delta T_{\text{l}}=\int_{0}^{\bar{\varepsilon}_{O}} \frac{Q_{\text{c}}\text{d}\bar{\varepsilon}}{\lambda_{\text{l}}A} \\ =\frac{Q_{\text{c}}}{\lambda_{\text{l}}}\int_{0}^{\theta} \frac{2\left(1+\cos\alpha\right)}{\pi d^{2}}\frac{d}{4\left(1+\cos\alpha\right)}\text{d}\alpha\\ =\frac{Q_{\text{c}}}{2\pi d\lambda_{\text{l}}}\int_{0}^{\theta} 1\text{d}\alpha\\ =\frac{Q_{\text{c}}}{2\pi d\lambda_{\text{l}}}\left.\alpha\right|_{0}^{\theta} \\ =\frac{Q_{\text{c}}\theta}{2\pi d\lambda_{\text{l}}} \\ =\frac{Q_{\text{c}}\theta\tan\frac{\theta}{2}}{4\pi h\lambda_{\text{l}}} \end{matrix}$$

Note that $d=2r_{\text{I}}\sin\theta=\frac{2h\sin\theta}{1-\cos\theta}=\frac{2h}{\tan\frac{\theta}{2}}$.
